# Atraumatic Bilateral Patella Fracture in Middle-Aged Female: A Rare Case Report

**DOI:** 10.1155/2024/6661957

**Published:** 2024-01-23

**Authors:** Ishwor Ghimire Padhya, Saral Lamichhane, Pramod Devkota, Praful Gurung, Pragya Aryal

**Affiliations:** ^1^Patan Academy of Health Sciences, Patan, Nepal; ^2^Gandaki Medical College, Pokhara, Nepal; ^3^Amppipal Hospital, Palungtar, Nepal

## Abstract

The patella is the largest sesamoid bone in the body and an important structure of the extensor apparatus which is under undue stress during flexion and extension of the knee. Bilateral fracture of the patella without trauma is a very rare event and may be multifactorial without a single cause. A repetitive stress reaction in a previously predisposed bone can be an important cause. We report a case of a 45-year-old female with a nontraumatic bilateral transverse patella fracture with loss of extensor mechanism. A stable surgical fixation for such a displaced fracture with a good rehabilitation program can lead to a good functional outcome.

## 1. Introduction

Bilateral patellar fracture is a rare entity, first described in 1943 by Muller. The mechanism of bilateral patella fracture can be traumatic or atraumatic; the atraumatic variant is much rarer than the traumatic one. Not a single case of stress fracture of the patella was reported among 320 cases of stress fractures in athletes [1] and also none in another study including 105 elderly patients with stress fractures [2]. One study, however, reported 18 cases of stress fracture of the patella in 2018 [3]. Being the largest sesamoid bone of the body and an important structure in the extensor apparatus of the knee, it can bear force as much as seven times the body weight [4]. Patella is richly supplied by blood vessels through a ring that runs from the distal to the proximal direction, and this rich vascularity may be the reason for such rarity of the stress fracture in the patella [3, 5]. We hereby present a rare case of a 45-year-old female with an atraumatic bilateral patella fracture.

## 2. Case Presentation

A 45-year-old female, farmer by occupation, presented to the emergency department in our tertiary care hospital with a chief complaint of severe pain in the anterior aspect of the bilateral knees for two hours and a history of mild, nonprogressive pain in the anterior part of the bilateral knees for two months. She was doing her household work and was about to stand from sitting position with firewood in her hand when she suddenly started having severe pain in her bilateral knees. She was not able to bear weight. There was no history of significant trauma, swelling, fever, or localized rise in temperature. She had no known chronic medical illnesses or previous surgical interventions. She was, however, diagnosed with essential hypertension during the hospital stay and was started on antihypertensive medications. She had a history of chronic alcohol consumption for the last 25 years and smoking for 20 years.

**Figure 1:**
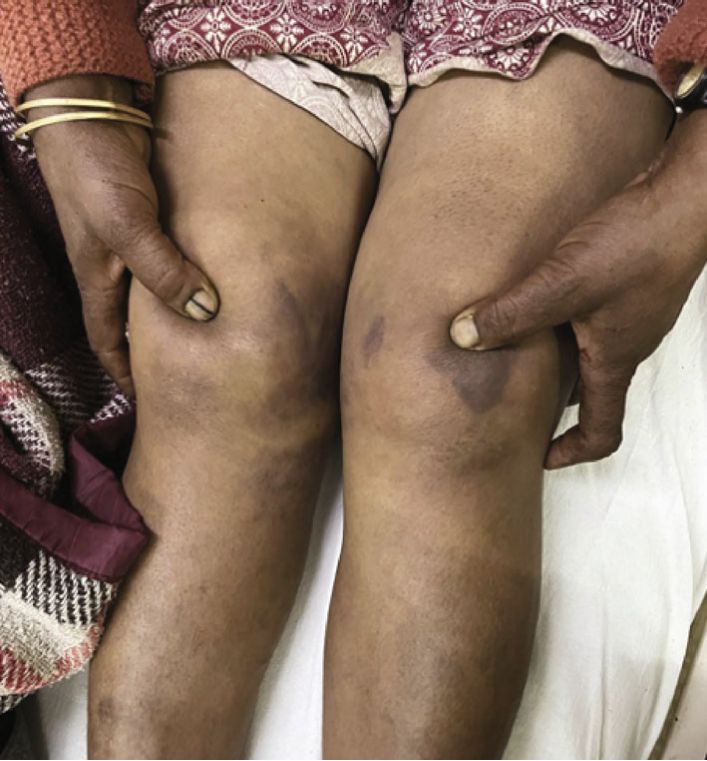
Patient showing sulcus sign.

On general examination, she appeared icteric and had bilateral pitting edema. On the local examination, she had tenderness and edema in the anterior aspect of the bilateral knee. The sulcus could be felt in the bilateral patella as shown in Figure 1. The extensor mechanism was lost.

**Figure 2:**
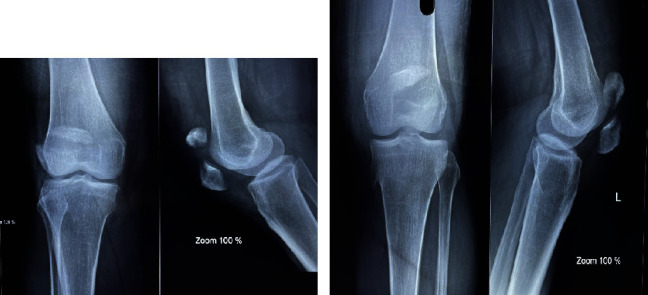
(a) X-ray of the right knee. (b) X-ray of the left knee with incidental osteochondroma.

Plain radiographs of the bilateral knees showed displaced transverse fracture of patella with displacement of more than 7 mm in bilateral knees (Figures 2(a) and 2(b)).

Laboratory investigations revealed normal hematological parameters, thyroid function tests, liver function tests, and renal function tests. Calcium levels were also within normal limits.

**Figure 3:**
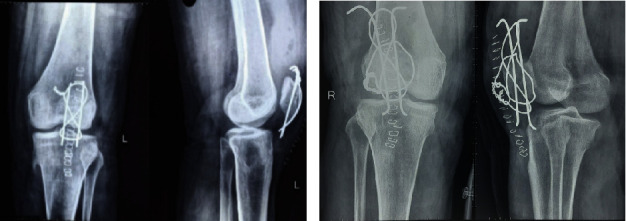
(a) Postop X-ray of the left patella. (b) Postop X-ray of the right patella.

The patient was hospitalized for surgical treatment (open reduction and internal fixation with tension band wiring with repair of extensor retinaculum) due to displacement of the fracture and loss of knee extension function. An anterior median incision was performed over the patella location, followed by a dissection of tissues to reach the fractured bone. The patellar tendon and retinaculum tears were observed. Patella fixation was performed using an “eight-pattern” with stainless wire looped through two parallel k wires. The extensor retinaculum was repaired with Vicryl (polyglactin) sutures. The postoperative plain radiographs of bilateral knees are shown in Figures 3(a) and 3(b).

**Figure 4:**
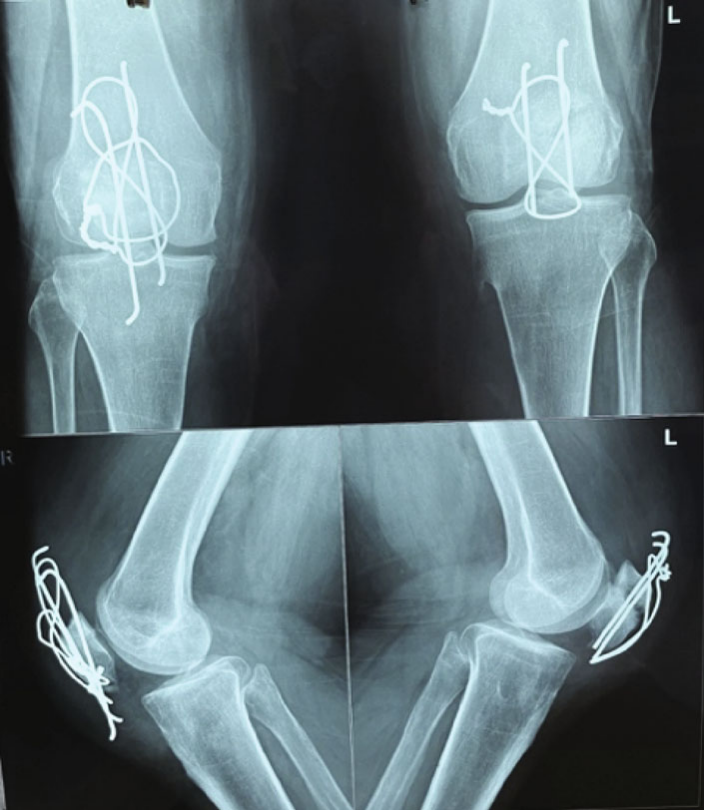
B/L knees after fracture consolidation.

In the postoperative period, the patient complained mild to moderate pain at the incision site, which was managed with usual analgesic drugs. She also received care from the physiotherapy team. During the next six weeks, the patient continued physiotherapy without standing or walking. Walking was then reintroduced progressively. Gait rehabilitation and progressive resistance exercises for muscle strengthening were also introduced gradually. Almost two and half months after surgery, the patient recovered with a normal gait and full range of motion in both knees and returned to her daily activities with some episodic hardware irritation. The radiograph of bilateral knees after consolidation is shown in Figure 4.

## 3. Discussion

As bilateral patella fracture due to indirect mechanism (atraumatic) is a rare entity, the exact cause and pathogenesis are yet to be identified. It has been mostly described as a stress fracture with multifactorial causes. One interesting case with the initial involvement of an incomplete fracture on the tension side of the right patella healed uneventfully, whereas the left progressed to a complete fracture with mild displacement [6]. Other causes like osteoporosis, renal diseases, hyperparathyroidism, vitamin D deficiency, nicotine use, and chronic alcohol consumption have also been implicated in the progression to stress fracture [2].

One study among 2634 military recruits with stress fractures found that the fracture cases had lower mean serum vitamin D levels at the time of entry to training compared with controls [7]. Deficiency of vitamin D due to reduced sun exposure leads to decreased levels of calcium and increased parathyroid levels [8]. A balanced calcium and vitamin D metabolism seems to be of paramount importance for stress fracture prevention in elderly patients [2]. Alcohol has been considered another risk factor for a stress fracture of a bone. A meta-analysis from 11 studies including 46,916 cases showed an evidence that increased alcohol consumption is associated with a higher risk of osteoporotic hip fracture; however, the role of alcohol at lower doses is uncertain [9].

In our case, the patient was a premenopausal female with risk factors of smoking and chronic alcoholism to weaken her bones. Her mild pain in bilateral knees for two months may have been due to a stress reaction in the patella. In a predisposed patella, the fracture might have occurred when she was about to stand from a sitting position as there is an enormous increase in pressure in the terminal 15 degrees of extension. Otherwise, she had a normal calcium level (indirect evidence of normal vitamin D metabolism) and normal renal parameters ruling out those causes.

The initial investigation for stress fracture is plain X-ray in AP and lateral view, but it has low sensitivity. A high degree of suspicion is required in the initial stage. MRI is the most sensitive and specific imaging test for diagnosing stress fractures [10]. Computed tomography (CT) scan also provides exquisitely fine osseous detail but should be reserved only for specific indications because it involves ionizing radiation [11]. However, in the later stage of the fractures, as in our case where there was a displaced fracture, an X-ray may be sufficient for diagnosis.

Management of patellar fracture can be conservative or operative. Nondisplaced, closed patellar fractures, or fractures with less than 2 mm articular steps can be successfully treated conservatively [12]. Surgical treatment is recommended in fractures with any of the following features: articular step‐off > 2 mm and >3 mm of fracture displacement, open fractures, and displaced fractures affecting the extensor mechanism [12, 13]. Among the various surgical options available for noncomminuted transverse patellar fractures (2-part), modified anterior tension band wiring is the treatment of choice due to its biomechanical superiority [13]. Care team including proper physical therapy is important in recovery.

## 4. Conclusion

Bilateral stress fracture of the patella is a very rare condition with no single identifiable cause but probably related to increased stress in a predisposed bone. A high degree of suspicion is required for the diagnosis of this condition. If indicated, surgical treatment with open reduction and internal fixation along with the repair of the extensor retinaculum can lead to good functional outcome in the patient, with a help of a multidisciplinary team. Further studies are warranted for better understanding of the causative factors and management of such condition.

## Data Availability

The datasets used and analyzed during the current study are available from the corresponding author upon reasonable request.
